# Illness perceptions in relation to experiences of contemporary cancer care settings among colorectal cancer survivors and their partners

**DOI:** 10.3402/qhw.v9.23581

**Published:** 2014-07-22

**Authors:** Ann-Caroline Johansson, Malin Axelsson, Ina Berndtsson, Eva Brink

**Affiliations:** 1 Department of Nursing, Health and Culture, University West, Trollhättan, Sweden; 2 Institute of Health and Care Sciences, University of Gothenburg, Gothenburg, Sweden; 3 Department of Care Science, Faculty of Health and Society, Malmö University, Sweden

**Keywords:** Cancer care, illness perception, colorectal cancer, grounded theory, nursing, partners

## Abstract

Illness is constituted by subjective experiences of symptoms and their psychosocial consequences. Illness perceptions concern people's lay beliefs about understandings and interpretation of a disease and expectations as to disease outcome. Our knowledge about illness perceptions and coping in relation to the cancer care context among persons with colorectal cancer (CRC) and their partners is incomplete. The aim of the present study was to explore illness perceptions in relation to contemporary cancer care settings among CRC survivors and partners. The present research focused on illness rather than disease, implying that personal experiences are central to the methodology. The grounded theory method used is that presented by Kathy Charmaz. The present results explore illness perceptions in the early recovery phase after being diagnosed and treated for cancer in a contemporary cancer care setting. The core category *outlook on the cancer diagnosis when quickly informed, treated, and discharged* illustrates the illness perceptions of survivors and partners as well as the environment in which they were found. The cancer care environment is presented in the conceptual category *experiencing contemporary cancer care settings*. Receiving treatment quickly and without waiting was a positive experience for both partners and survivors; however partners experienced the information as massive and as causing concern. The period after discharge was being marked by uncertainty and loneliness, and partners tended to experience non-continuity in care as more problematic than the survivor did. The results showed different illness perceptions and a mismatch between illness perceptions among survivors and partners, presented in the conceptual category *outlook on the cancer diagnosis*. One illness perception, here presented among partners, focused on seeing the cancer diagnosis as a permanent life-changing event. The other illness perception, here presented among survivors, concentrated on leaving the cancer diagnosis behind and moving forward. The importance of illness perceptions among survivors, and the differences in illness perceptions between survivors and partners, should be recognized by healthcare professionals to achieve the goals of person-centered contemporary cancer care.

Colorectal cancer (CRC) is the third most common cancer in industrialized countries, among both females and males (Ferlay et al., 2010). Uncomplicated CRC is treated with surgery and the hospital time is 3–10 days, with radiation and chemotherapy as additional treatments (Varadhan et al., 2010). Diagnosis, treatments and side effects, reactions of family and friends, follow-up, recurrence uncertainties during recovery, and rehabilitation all cause stress in a person with CRC. This can lead to negative somatic effects as well as to psychological problems such as depression and anxiety about cancer relapse and not knowing what to anticipate about the future but also to psychosocial problems such as reduced social activity because of feelings of being treated differently because of the disease or simply due to the obstacle of needing to have constant access to toilet facilities (Dunn et al., 2006).

The relation and the dynamics between couples are influenced by a cancer diagnosis. Partners often feel obliged to be encouraging and persons with cancer often show a protective side, keeping their emotions out of reach from the partner (Emslie et al., 2009; Houldin, 2007). In addition, partners have reported added emotional stress compared to the persons with cancer have reported added emotional stress compared to the persons with cancer (Northouse, Mood, Templin, Mellon, & George, 2000). People close to persons with CRC have in fact shown to be one of the worst affected groups regarding increased risk of developing mental illness and cardiovascular disease (Sjövall et al., 2009).

Whereas disease concerns pathology and biomedical disease markers, illness is constituted by subjective experiences of symptoms and psychosocial consequences (Bhugra & Malhi, 2013; Eisenberg, 1977). The present focus on illness rather than disease implies that personal experiences are central to the methodology. Also, persons who are former cancer patients are referred to as survivors. The concept of cancer survivorship refers to a process that begins with a diagnosis, which in turn involves individual features of uncertainty and positive and negative aspects, all with consequences for health. This concept concerns the process from diagnosis and disease through treatment to health and survivorship and promotes a holistic view of individuals (Doyle, 2008), which is in harmony with the person concept in person-centered care that emphasizes a person's illness experiences and view of the life situation (Ekman et al., 2011). In addition, letting partners have a voice in care decisions may enhance the successfulness of person-centered care (Boise & White, 2004).

Illness perceptions concern people's lay beliefs about, understanding, and interpretation of a disease and expectations as to disease outcome. In parallel with this cognitive process there is an emotional response. Based on the cognitive and emotional presentation of the illness, a coping response is shaped and carried out (Leventhal, Nerenz, & Steele, 1984; Leventhal et al., 1997). The commonsense model of illness representations is a central model in Leventhal's Self-Regulation Theory. The model focuses on how implicit illness beliefs shape coping and adjustment (Leventhal, Meyer, & Nerenz, 1980). The self-regulation theory and the commonsense model of illness representations provide a structure for understanding individual variance in representations to illness. According to this theory, illness can be conceived of as a cyclical process of interpretation, coping, and evaluation.

Our knowledge about illness perceptions among persons with CRC and their partners is incomplete. Findings presented by Rosenfeld (2006) suggest that persons with CRC expect cancer to be acute and short lasting. Otherwise illness perceptions in persons with CRC have been studied in the context of genetic screening (Van Oostrom et al., 2007) and personality (Mols, Denollet, Kaptein, Reemst, & Thong, 2012).

There are findings that show the importance of the physical and psychosocial environment of cancer care settings for persons treated for cancer (Browall, Koinberg, Falk, & Wijk, 2013; Edvardsson, Sandman, & Rasmussen, 2006). Based on their findings, Browall et al. (2013) suggested that the psychosocial environment is of greater importance than the physical environment for persons with cancer. Nevertheless, according to Edvardsson et al. (2006), the physical environment is an important symbol of care, and is charged with value. Neglecting the physical environment signals neglecting people, and vice versa. To our knowledge, there are no previous studies focusing on CRC care settings and illness perceptions in persons with CRC and their partners. Therefore, the aim of the present study was to explore illness perceptions in relation to experienced contemporary cancer care settings among CRC survivors and partners.

## Methods

### Design

The present study was conducted using grounded theory and a methodology based on symbolic interactionism. The methodological perspective is social constructivist, which recognizes social life as being processual in nature. This perspective sees people as existing and acting within a social environment that they influence and are influenced by. The grounded theory method used, in accordance with the methodological perspective of the study, is that presented by Kathy Charmaz (2006).

### Participants and setting

The study was approved by the Regional Ethical Review Board of Gothenburg (Reg. no. 753-10). Participants (survivors) were recruited from a county hospital in western Sweden. CRC survivors who participated in a survey study were contacted by phone. Survivors were informed about the study, invited to participate, and asked for permission to contact their partner. Those interested received a written letter of information and a written consent form was returned by the survivor and partner prior to the interview. In total 18 persons participated (nine survivors and nine partners). Four survivors were interviewed together with their partner. In the other cases, five survivors were interviewed without participation on the part of their partners, who had declined participation. The survivors varied in age between 61 and 85 years and the partners between 58 and 87 years. The survivors were three males and six females and the partners were three males and six females. Characteristics of the participants are shown in Table I.

**Table I:** Characteristics of participants.

Interview	Participants^a^	Sex	Age	Partnership^b^	Occupation^c^	Diagnosis^d^	Stoma^e^	Chemo^f^	Radiation^g^
1	S	F	75	M	R	R	Y	N	N
	P	M	75	M	R				
2	S	F	75	L	R	R	N	N	N
	P	M	87	L	R				
3	S	F	74	M	R	R	Y	N	Y
	P	M	77	M	R				
4	S	M	85	C	R	C	N	N	N
	P	F	75	C	R				
5	P	F	70	M	R				
6	S	M	71	M	R	R	Y	N	N
7	P	F	58	M	W				
8	P	F	65	M	W				
9	P	F	64	L	W				
10	P	F	64	M	W				
11	S	F	61	M	W	C	N	N	N
12	S	F	79	M	R	R	Y	N	N
13	S	M	85	L	R	C	N	N	N
14	S	F	68	M	R	R	Y	N	N

a Participant: S=Survivor; P=Partner.

b Partnership: M=Married; C=Cohabitant; L=Living apart.

c Occupation: R=Retired; W=Working.

d Diagnosis: R=cancer recti; C=cancer coli.

e Stoma: Y=Yes; N=No.

f Chemo: Y=Yes; N=No.

g Radiation: Y=Yes; N=No.

### Data collection

Survivors and partners were interviewed separately, except in four cases when the survivor and partner were interviewed together. All participants were interviewed by the first author. The interviews were conducted at a place chosen by the participants: University West (*n=*3), neutral location (*n=*6), or the participant's home (*n=*9). All interviews were completed during the 10-month period from October 2011 to July 2012. All interviews were performed 3–10 months after surgery; this period has been found to be of importance in previous research showing a decrease in quality of life over time among survivors aged >60 (Arndt, Merx, Stegmaier, Ziegler, & Brenner, 2004). Interviews were conducted by asking all participants the same opening question: *Can you describe an ordinary day and what it is like for you?* This opening question was chosen because it made the transition to the sensitive topic of the cancer disease easier for survivors and partners. The opening question was followed by open-ended questions on illness and healthcare experience. For instance, survivors were asked: *What do you think about the disease today? What do you think about the healthcare and treatment given?* Corresponding questions to partners could be: *What do you think about your partner's disease*? *What do you think about the healthcare and treatment given to your partner?* Probing questions were posed, such as: *In what way has this affected you?*

### Data analysis

Interviews were transcribed, then coded actively and line by line using Nvivo (Edhlund, 2011). Analysis was performed in parallel with interviewing and memo writing, which allowed theoretical sampling by developing and changing interview questions as new properties of the categories emerged and new experiences needed to be covered. Constant comparative methods were used from the beginning to the end of the analysis, and the development and richness of properties discovered were described in memos. Memos became the framework for focused coding. Interaction with the data and the use of sensitizing concepts were then the starting point for focused codes and ideas, at the same time as rethinking was done by making comparisons and following leads. Extensive memo writing was helpful at this point, the aim being to maintain theoretical sensitivity and not force the data to match preconceived ideas (Charmaz, 2006). Some focused codes synthesized large sections of the data and were raised to the level of categories. Properties of categories were consistently described, compared, and developed. Relationships between categories were further clarified by clustering, which enabled the later development of conceptual categories to synthesize the properties of several categories. At the end of the analysis, the core category had taken shape, and the analysis was considered to be complete, as the researchers were no longer able to make progress.

## Results

The present results explore illness perceptions in the early recovery phase after being diagnosed and treated for cancer in an experienced contemporary cancer care setting. The core category *outlook on the cancer diagnosis when quickly informed, treated, and discharged* illustrates the illness perceptions of survivors and partners as well as the environment in which they were found. The cancer care environment is presented in the conceptual category *experiencing contemporary cancer care settings*. Receiving treatment quickly and without waiting was a positive experience for both partners and survivors; however partners experienced the information as massive and as causing concern. The time after discharge was also being marked by uncertainty and loneliness, and partners tended to experience non-continuity in care as more problematic than the survivor did. The different illness perceptions found are presented in the conceptual category *outlook on the cancer diagnosis*. The different illness perceptions seen in the present findings showed a mismatch between survivors and partners. One illness perception, here presented among partners, focused on seeing the cancer diagnosis as a permanent life-changing event including an active information-seeking behavior and a focus on the cancer word. The other illness perception, here presented among survivors, concentrated on leaving the cancer diagnosis behind and moving forward, involving biding one's time and focusing on words that did not confirm or refute the cancer disease. Development of the core category—*outlook on the cancer diagnosis when quickly informed, treated, and discharged*—and the conceptual categories are presented in Table II.

**Table II:** Development of the core category and the conceptual categories.

Outlook on the cancer diagnosis when quickly informed, treated, and discharged			

Conceptual category	Experiencing contemporary cancer care settings	Conceptual category	Outlook on the cancer diagnosis
Category	Experiencing compressed time	Category	Seeing the cancer diagnosis
Subcategory	Short timeline	Subcategory	Having a direct outlook on information
Focused code	Experiencing flow	Focused code	Being resolute
	Treated before knowing it		Information seeking
			Patient guardian
			Life-changing disease
Subcategory	Being in a burst of information	Subcategory	Focusing on the value of the cancer word
Focused code	Experiencing a huge amount of information	Focused code	Selecting value charged interpretation
	Fear of forgetting details		Recognizing the seriousness
	Taking charge		Needing to know
Category	Being left in echoing silence	Category	Leaving the cancer diagnosis behind
Subcategory	Not knowing what to expect	Subcategory	Having a submissive outlook on information
Focused code	Information insecurity	Focused code	Being content
	Experiencing unpredictability		Handing oneself over
			Creating distance to information
Subcategory	Lacking continuity	Subcategory	Focusing on value neutral words
Focused code	Being sent back and forth	Focused code	Neither confirming nor refuting diagnosis
	Feelings of being abandoned		Having an uncomplicated outlook on illness

### Experiencing contemporary cancer care settings

This conceptual category contains the participants’ experiences of the cancer care environment, including information and communicational settings. Receiving treatment quickly and without waiting was a positive experience for both partners and survivors. However, partners describe contemporary cancer care as an environment where partners sometimes felt so overwhelmed by the information that they were concerned about forgetting, feeling they had to take charge over the care situation. Survivors were not concerned in the same way about the huge amount of information given. The time after discharge was experienced as a time when feelings of uncertainty and loneliness were present. The different illness perceptions of survivors and partners were found in their experiences after the diagnosis and treatment for CRC.

### Experiencing compressed time

A major issue surrounding expressed experiences of the hospital stay was the length of time during which these experiences took place. *Experiencing compressed time* was a category developed from the subcategories *short timeline* and *being in a burst of information*.

#### Short timeline

Receiving treatment quickly and without waiting was a positive experience for both partners and survivors. The participants described the experience of a short waiting time as effective and smooth.

It went quickly. Two months from the first doctor's appointment until my operation. And during the doctor's appointment when I found out the doctor acted right away and arranged times for different x-rays. It flowed really well. (Interview 14, survivor, man)

It all went so fast from when we found out until he was scheduled for surgery and to get a colostomy, two weeks or a month. And that was really good too. So it really went quickly. (Interview 5, partner, woman)

The participants’ statements about their experiences from the first doctor's appointment to the hospital stay described these experiences as rapid events, where every medical situation sped by, almost in the blink of an eye.

I hardly had time to blink … and it was off to the regional hospital and radiation for 5 days and then home on Friday, at home on Saturday and Sunday and then on Monday up to the county hospital for surgery on the 20th of December and them home on Christmas Day …. (Interview 3, survivor, woman)

#### Being in a burst of information

Partners described the information given during medical appointments and at hospital stays as mostly verbal, extensive, massive, and difficult to grasp and remember.

The only thing I know is that I thought there was an incredible amount of information. I don't know how many offices, or whatever they're called, we were at … I know I thought many times that if only I knew how to take shorthand! … But I think all of them said to just call if we had questions. (Interview 5, partner, woman)

The large amount of verbal information caused partners to be concerned about forgetting details. Missing written information or delays in written information, such as letters with new appointment times, therefore required energy and a certain amount of pressure on the healthcare system.

When you know there will be examinations and treatments … and that getting appointments and written information has taken time, which it really has. We've had to push pretty hard there. (Interview 7, partner, woman)

Keeping track of new appointments, coordinating care and information was a way of taking charge of the situation. One aspect of taking charge that was described as problematic was the uncertainty about whom to contact when clarification was needed. The results showed that taking charge of the situation and remembering the information given were experienced as more burdensome by partners.

### Being left in echoing silence

Articulated experiences from the period close to discharge, during, and after discharge from hospital, included feelings of unpredictability, informational insecurity, and feelings of being abandoned after medical care. Being left in echoing silence was a category developed from the subcategories *not knowing what to expect* and *lacking continuity*.

#### Not knowing what to expect

The participants’ statements contained elements of unpredictability and informational insecurity concerning their discharge, for instance, getting different information from different healthcare professionals, leaving the soon-to-be-former patient and partner not knowing what to expect.

Things were a bit uncertain when I was going to go home, because one nurse said you probably have to stay here a few days. But then when the doctor came—a young female doctor and a nurse. Just the two of them, Well, you get to go home they said, and that was the same day … If you have any problems just contact us they said. (Interview 4, survivor, man)

#### Lacking continuity

Participants also described situations of being sent back and forth in the healthcare system. No one cared or took the time to explain. An important aspect of “lacking continuity” involved partners’ feelings of being abandoned and left on their own.

… he was discharged on Monday and they said that we should go to our primary healthcare center on Friday and have the catheter removed …. We went there and they took the catheter out and it didn't work …. So we had to go to the emergency room in the middle of the night and tell them nothing was coming out … there were several things like that … after being discharged there was nobody who checked how things were going. They were very quick about all the blood tests and other tests that had to be taken. But nobody ever sits down and talks to the person who is going through all this … nobody! (Interview 9, partner, woman)

He was at a doctor's appointment and I was there too and then the doctor said we'll see you again in a year … then that was it, and I don't have anybody I can talk to … I don't know who I would call. (Interview 6, partner, woman)

The results showed that partners experienced non-continuity in care as more problematic than the survivors did. Partners’ feelings of being abandoned seem to be strengthened by the fact that they had no specific healthcare professional to contact if needed; they just knew that they should contact the healthcare system if problems occurred.

### Outlook on the cancer diagnosis

This conceptual category contains different beliefs and thoughts about the cancer illness and its consequences among the participants, exposing diverse understandings and interpretations of the illness and the recovery period. In the cancer care environment, which was experienced as rapid and including a huge amount of information, two different illness perceptions were present but unnoticed by healthcare professionals. They are described in the category *seeing the cancer diagnosis*, including the subcategories *having a direct outlook on information* and *focusing on the value of the cancer word*, and the category *leaving the cancer diagnosis behind*, including the subcategories *having a submissive outlook on information* and *focusing on value-neutral words*.

### Seeing the cancer diagnosis

The category *seeing the cancer diagnosis* was developed from the subcategories *having a direct outlook on information* and *focusing on the value of the cancer word*. Seeing the cancer diagnosis implies a willingness to accept the seriousness of the concept of cancer by embracing the meaning brought about by the concept. It means that the participants were prepared to face the severe consequences of the cancer disease and embraced the possibility of relapse and that the survivor's health might never be what it had been prior to the cancer. The cancer disease was considered a permanent, life-changing event that would be present and influence life from now on.

… Because that's what I experienced that he expected that now I'm going to be completely normal again … maybe he never will be. (Interview 8, partner, woman)

This life-changing perception was more frequent among partners than survivors. When partners had this perception in solitude and did not share it with the survivor, frustration and difficulties emerged in relation to communication. Partners saw a disease reality they wished to communicate to the survivor and to healthcare professionals, but they did not know how to do this.

He had to have an examination … then we talked after every … but he showed very clearly and he actually said I don't feel sick … and then I can't say well you should – you have cancer! (Interview 8, partner, woman)

Soon he'll go to one of these you know 1-year check-ups. And I feel like I'm getting more and more stressed out … of course he has to admit he's tired, but he connects it to his work and I connect it to how he was last year …. (Interview 6, partner, woman)

#### Having a direct outlook on information

Which views or actions the participant had or took in relation to information was interpreted as part of illness perception, and connected to outlook on the cancer diagnosis. This direct approach among participants is focused on finding answers and to understand information properly. Envisioning the cancer disease as a permanent, life-changing event meant that this active information-seeking behavior involved elements of frustration among partners in relation to the information provided by healthcare professionals.

And it's happened frequently that I'm sitting there next to him and they explain something to him and he says yes, and then we get outside the door and he doesn't understand anything. So really they have to consider that the patient is in shock and confused and doesn't understand things. (Interview 9, partner, woman)

#### Focusing on the value of the cancer word

Participants’ interpretation of verbal information and lay understandings of the disease concepts used by healthcare professionals differed among the participants, and this variation was considered to be associated with approaches to information. It was understood as a way to emphasize that cancer is a disease that will always be present in life from this point forward. This focus was more commonly expressed among partners. When survivor and partner did not share the same focus, the partner felt torn between wanting to use the cancer word and wanting to protect the survivor.

… Sometimes I say the word cancer straight out. No I didn't have it he says, it could have developed into it but it wasn't. And then I feel bad because I've said a word or claimed something he doesn't agree with. (Interview 6, partner, woman)

Focusing on the value of the cancer concept was interpreted as being connected to having a direct outlook on information through the need to know, and the need for explicit information, which was highlighted when partners perceived a difference in focus as being caused by improper word choice or inadequate communication about the cancer disease on the part of healthcare professionals.

… Then they said that this is a tumor and they said we'll get it quickly …. But now he says **tumor and not cancer** so there is something there. (Interview 8, partner, woman)

They told him you have a little thingy; they told him at one of the first appointments … I think that expression was wrong, they should have explained so that they could see that he got it and then maybe lightened things up a bit. But this funny word and he's like the prankster and doesn't get it then that's not right …. (Interview 6, partner, woman)

### Leaving the cancer diagnosis behind

The category *leaving the cancer diagnosis behind* was developed from the subcategories *having a submissive outlook on information* and *focusing on value-neutral words*. The participants’ statements contained elements of minimizing the seriousness of the disease.

It's just that people get so terribly worked up about cancer. It's like it was the end of the world. Of course many people have it, but they've made such progress with cancer research. Many people get so scared …. I'm really pleased, everything went so well. I haven't had problems with anything …. (Interview 3, survivor, woman)

But the participants’ statements also contained a stance focused on moving forward. Leaving the cancer diagnosis behind revealed a perception that reduced the seriousness of the disease by decreasing the impact connected with the concept of cancer. The cancer disease was considered to be acute and transitory in nature: When treatment was over, so was the cancer disease. This was clarified when participants focused on leaving the illness in the past and instead emphasized experiencing health in the present.

I don't think about it so often. I don't really it's almost forgotten. (Interview 4, survivor, woman)

… And I don't experience it like I have an illness. (Interview 11, survivor, woman)

Leaving the cancer diagnosis behind was frequent among survivors. The partners, however, often noticed this perception even if it was not shared by them. Partners sometimes reacted to the fact that the illness perception of the survivor was not consistent with their own view of the reality of the disease, which sometimes led to confusion. Their statements revealed their belief that the survivor somehow separated the disease from the self, thus creating a distance.

… well it just hasn't been that easy! … like he describes it and like I interpret what he says, and I think also when he talks to other people. It's as if it was something that was part of his body, but didn't really have anything to do with him. (Interview 7, partner, woman)

The message from the quote below is about a reality of disease and health that is not shared by the survivor and partner. This female partner talked about the difference between how she viewed the disease and health of her husband compared to the view her husband communicated.

… he's as healthy as a horse … you see, that's what he says. (Interview 6, partner, woman)

#### Having a submissive outlook on information

This outlook on information constituted trust and humility in relation to healthcare professionals’ knowledge and ability to give the required information when needed. This submissive informational approach is interpreted as being part of *leaving the cancer diagnosis behind*, where trusting in healthcare professionals’ and the healthcare system's ability to help one recover from this acute and transitory disease is absolute. There is, thus, no need to dig deeper and know more than what one has been told, instead the emphasis is on biding one's time, having faith and placing oneself in the hands of professionals.

The doctor does what he does and the gals do what they do and the radiation guy does his thing and then we just hope for the best. It's like the doctors asking me before the operation if I had any questions. No I said, I wouldn't know what to ask …. (Interview 3, survivor, woman)

This cautious outlook on information was held by survivors, and involved seeing information as something that was provided by professionals and cleared up with time and patience, not by asking questions.

I haven't received an appointment anyway …. So I hope it will be before summer anyway …. (Interview 2, survivor, woman)

Lack of continuity was therefore faced with equanimity among those who had a submissive outlook on information.

… since then she hasn't been here, my nurse. No—once … but she was going to try to come another time … so I had a new one this time and the previous time a new one too. But they're all equally good so it doesn't matter. (Interview 1, survivor, woman)

When the survivor had a submissive outlook on information, it often led to frustration in the partner.

He's going to have a yearly check-up soon and I just want to know why all the time. I really want him to find out what they're going to do. Because I ask what are they going to do? ‘uh I don't know’ what does it say on the papers? ‘uh I haven't looked’ and then I think well do it now. (Interview 8, partner, woman)

#### Focusing on value-neutral words

Focusing on a word that was neutral in value and that did not confirm or refute the cancer disease was calming and created hope. Focusing on value-neutral words was interpreted as being connected to the subcategory *having a submissive outlook on information* by shielding oneself from unnecessary information and having faith.

Then the doctor who examined me said, he said he'd seen worse. So I think you've come in in time and we can operate on **it**. And his words gave me great hope they calmed me. (Interview 10, survivor, woman)

The results show that it is survivors who reduced the value of the cancer concept by talking about the cancer disease using words like it, growth, alteration, and tumor, without clarifying that is was a cancerous tumor. However this omission of the cancer word was often noticed by partners.

He (her husband) didn't say cancer instead he said, I have a tumor in my intestines that they're going to remove …. (Interview 7, partner, woman)

## Discussion

The present results showed different illness perceptions of survivors and partners and the experienced cancer care settings in which they were found. One illness perception, more common among partners than survivors, focused on seeing the cancer diagnosis by embracing the meaning brought about by the concept, and being prepared for severe and long-lasting consequences. The other, which was more frequent among survivors, concentrated on leaving the cancer diagnosis behind by reducing the seriousness and decreasing the impact connected with the concept of a cancer diagnosis. The different illness perceptions and the different perspectives among partners and survivors are, however, interchangeable and situation-dependent, in that partners and survivors may also be in different phases of the illness trajectory. Nevertheless, the study offers some guidance concerning what illness perceptions might look like in contemporary cancer care settings.

The illness perception covered by the category “seeing the cancer diagnosis” involves emphasis on the value of the cancer concept and its serious and life-changing properties. This perception could indicate a stance of acceptance, but it could equally indicate catastrophic and worst-case-scenario thinking. Interestingly, this perception was more present among partners. Previous research has shown that negative appraisal of the ailing person's illness, such as seeing the cancer and treatment as more severe and stressful or having feelings of uncertainty and despair, can contribute to distress and development of affective disorders, especially among female partners (Pitceathly & Maguire, 2003). In addition, research by Sjövall et al. (2009) has shown that close relatives of persons with CRC are at higher risk of developing mental illness and cardiovascular disease. The power of partners’ perceptions should therefore not be underestimated. Research has in fact suggested that the illness perceptions of persons close to the individual with cancer also influence the ailing person's coping behavior (Lobban, Barrowclough, & Jones, 2003; Sterba & DeVellis, 2009). The present study did not focus on coping, but according to the self-regulation theory and the commonsense model of illness representations, illness perception is what underlies the coping process (Leventhal et al., 1984). From a coping perspective, one possible explanation for the opposite outlooks on information held by partners and survivors, and especially for partners’ more active involvement in the care, is provided by Nolan, Grant, and Keady (1996), who suggested that involvement is an important coping strategy that brings satisfaction to the partner.

From a caring perspective, the rapid movement through the healthcare system from admission to discharge may hamper the ailing person's understanding of the illness. People need time to understand any situation, not least a life-threatening one. The importance of this short time, particularly short time for recovery, has previously been suggested to contribute to CRC survivors’ difficulty in understanding that they had in fact been treated for cancer (Ohlsson-Nevo, Andershed, Nilsson, & Anderzén-Carlsson, 2011). Contemporary cancer care settings may therefore contribute to this perception, presented among survivors through their leaving the cancer diagnosis behind. This perception could indicate tranquil acceptance, but it could equally indicate difficulties in comprehending the situation. If the latter is the case, it could be problematic for persons who need time and support to reflect and to take in information. The perception among partners, presented through their seeing the cancer diagnosis regardless of whether their view is realistic or slightly catastrophic, may reflect partners’ reaction to their perception that the cancer care setting is contributing to the ailing person's inability to understand the situation.

The present results emphasize that some aspects of the cancer care environment, such as continuity, coordination, and support after discharge, need improvements to better support the survivors and their partner. Thus, revising the content of information based on the different needs of survivors and their partners should be given priority. Research has already acknowledged that there are inconsistencies between the real informational needs of survivors and partners and what healthcare professionals believe their informational needs are (Degner, Davison, Sloan, & Mueller, 1998; Snyder et al., 2007). The explanation for why there sometimes is a poor fit between the information survivors and partners want and need and what they actually receive may lie in the cancer care settings of today. Contemporary cancer care is about saving lives through more rapid diagnosis, better treatment, and shorter waiting time. Improvement in these areas should of course always be the main goal. These cancer care settings may not, however, identify the treated person's illness perception, much less the partner's illness perception, or a possible mismatch between the two. At the same time, illness perceptions have been shown to be an important framework within which individuals interpret information (Llewellyn, McGurk, & Weinman, 2007). Thus, paying attention to illness perceptions among survivors and partners and adapting the information provided to the different perceptions may be beneficial for survivors and partners, as well as for the overall economy of the healthcare system. Information can be adapted simply by talking with the survivor and his or her partner, asking questions about their experiences, and exploring their thoughts about the disease and the future. If healthcare professionals provide information adapted to each individual's illness perception, the survivor and partner may be able to communicate with each other and with healthcare professionals in an easier and more efficient way. Using illness perceptions as a starting point for information and communication also gives an opportunity to gain access to the coping strategies used by the survivor and partner and to offer support when coping fails, which could prevent unnecessary psychological and psychosocial suffering in survivors and their partners.

Presenting information about a cancer disease is always a challenging task. It involves knowing not only how to present the information, but also which information to provide and to whom, as well as deciding how much information should be provided at the same time. The present results showed that providing large amounts of verbal information during a relatively short period of time may be problematic. Adjustment and coping processes influence the ability to take in information (Leydon et al., 2000; Mulcare et al., 2011), as does age, such that the older a person is, the less information should be given on one occasion (Ankem, 2006). Finally, information provision needs to be adapted not only to illness perceptions, but also to the individual's ability to take in information.

### Methodological considerations

The size of the present study makes the conceptual categories presented theoretically sufficient rather than saturated (Charmaz, 2006; Dey, 1999). The researchers began the literature review when the analysis was considered finished, as recommended for this method (Charmaz, 2006). Regular discussions during analysis and maintaining theoretical sensitivity were used to increase awareness of preconceived ideas. However, preunderstandings, adductive reasoning, and the use of sensitizing concepts in the abstraction phase mean that preconceived ideas have to some extent exerted an inevitable influence on the analysis.

The strength of the study design is that data have been used from individual interviews with survivors and partners whose significant others were not participants, as well as from joint couple interviews. Couple interviews provide insights into different experiences in the context of the relationship (Illingsworth, Forbat, Hubbard, & Kearney, 2010; Seymour, Dix, & Eardley, 1995). The relationship may function as an inhibitor, however, preventing open discussion on sensitive topics (Ohlsson-Nevo et al., 2011). Conducting individual interviews with survivors and partners whose significant others were not participants allows participants’ to express themselves openly and freely, without censorship. These different types of interviews thus gave access to data from three perspectives. Including multiple perspectives on the same event is known to be particularly beneficial in qualitative studies (Sandelowski, 2000).

One possible limitation is that the partners’ participation was dependent on the survivors’ consent. There could therefore have been partners who wished to participate but who were prevented from taking part by the person treated for CRC. Furthermore, consent may have predominantly been given to partners considered to be caring and involved. Another limitation of the present study concerning the recruitment is that women were overrepresented among survivors as well as among partners. Sex disparities may affect the present results, as previous findings on persons with CRC suggest that women may experience more negative emotional and physical consequences (McCaughan, Prue, Parahoo, McIlfatrick, & McKenna, 2011). Women may also have greater informational needs during the initial 9-month post-diagnosis than do male cancer survivors (Matsuyama, Kuhn, Molisani, & Wilson-Genderson, 2013). Female partners of cancer survivors have also been shown to be more vulnerable to emotional distress than their male partners (Pitceathly & Maguire, 2003).

## Conclusion

The present findings on different illness perceptions among partners and survivors—involving different interpretations of words and different outlooks on information as reflected in their experiences of contemporary cancer care settings—add new knowledge. Having a direct versus a submissive outlook on information is presented in the results as being part of illness perception. The present results emphasize that some aspects of the healthcare environment, such as information, continuity, coordination, and support after discharge, need to be improved to better support the survivor and partner. Healthcare professionals need to be aware of people's different illness perceptions and acknowledge these as a framework within which individuals interpret information. Illness perceptions should be used as a starting point for communication and additional information should be adapted to survivors’ and partners’ different needs. Finally, the importance of illness perceptions among survivors, and the differences in illness perceptions between survivors and partners, should be recognized by healthcare professionals if they are to achieve the goals of person-centered contemporary cancer care.
